# N_2_-rich fluid in the vein-type Yangjingou scheelite deposit, Yanbian, NE China

**DOI:** 10.1038/s41598-018-22227-7

**Published:** 2018-04-04

**Authors:** Yicun Wang, Keyong Wang, Yassa Konare

**Affiliations:** College of Earth Sciences, Jilin University, Changchun, 130061 China

## Abstract

Nearly pure N_2_ fluid inclusions (T_h_ (L) = −151~−168 °C; T_h_ (V) = ~150.3 °C) were identified in W-mineralized quartz veins from the Yangjingou scheelite deposit, in the eastern Yanbian area, NE China. Other fluid inclusion populations include N_2_-CO_2_, NaCl-H_2_O ± N_2_ and CO_2_ ± N_2_-NaCl-H_2_O, but no hydrocarbons were detected. The host rocks are part of the Wudaogou Group metamorphic series, which mainly consist of Ca-rich mica schist. Subhedral sulfide minerals occur in early disseminated W-mineralized quartz veins, or have partially replaced early scheelite. T_hN2_ and T_hN2-H2O_ indicate N_2_ fluid-trapping from 315 °C to 410 °C and from 80 MPa to 350 MPa. Oxygen and hydrogen isotopic data (δD = −74.9‰~−77‰, δ^18^O = 9.6‰~12‰, V-SMOW) suggest that the mineralizing fluids were composed of mixed magmatic and metamorphic water, N_2_-rich inclusions (δ^15^N = −0.5‰ to 1.4‰) indicate fluid-rock interaction with metamorphic rocks. The N_2_-rich fluid was closely associated with scheelite precipitation. During thermal decomposition under high oxygen fugacity conditions, which occurred synchronously with metamorphism and magmatic activity, large amounts of N_2_ were liberated from NH_4_^+^-micas, which then accumulated in the parent fluid of the quartz scheelite veins.

## Introduction

Nitrogen is the dominant constituent of the atmosphere and a key component in the biosphere. High-density CO_2_-N_2_ inclusions have been detected in high-grade metamorphic rocks from the upper mantle and lower crust (i.e., granulites and eclogites)^1–3^. In silicates, under conditions of elevated temperatures, low water activity, and high oxygen fugacity, N_2_ is favorably released from minerals containing ammonium (e.g., feldspar and mica)^4,5^.

Scheelite containing nitrogen is most commonly recognized to be traced from sedimentary rocks^6^. In sedimentary-type deposits, tungsten (W) is liberated as a result of exhalative processes, forming stratiform scheelite^6^, in which mineralizing fluids containing CH_4_ and N_2_ are generally attributed to *in situ* organic matter^6^. Several examples of scheelite deposits related to metamorphic hydrothermal fluids are reported to contain small amounts of N_2_^7,8^. These types of scheelite deposits are of interest to the geologist, because they are related to Au deposits^9^. Furthermore, Gibert^8^ found that the addition of only 5% N_2_ would decrease the solubility of scheelite in micaschists from 40 ppm to less than 3 ppm W, indicating that N_2_ may drive scheelite participation.

Nearly pure N_2_ (>88 mol%) was identified in fluid inclusions in W-mineralized quartz veins, which is absent of hydrocarbons. Previous studies have shown that NaCl-H_2_O and CO_2_ fluid inclusions are common in ore-forming fluids; however, the presence of N_2_-rich fluids in ore deposits is rarely reported. This study aims to identify the origin of the W-mineralizing fluid and the formation of the N_2_-rich fluid inclusions by combining petrography, Raman microspectroscopy, H, O and N isotope geochemistry, and microthermometry on fluid inclusions. Results from this study will improve our understanding of how N_2_ modulate scheelite mineralization.

## Geology of the Yangjingou scheelite deposit

**Figure 1:**
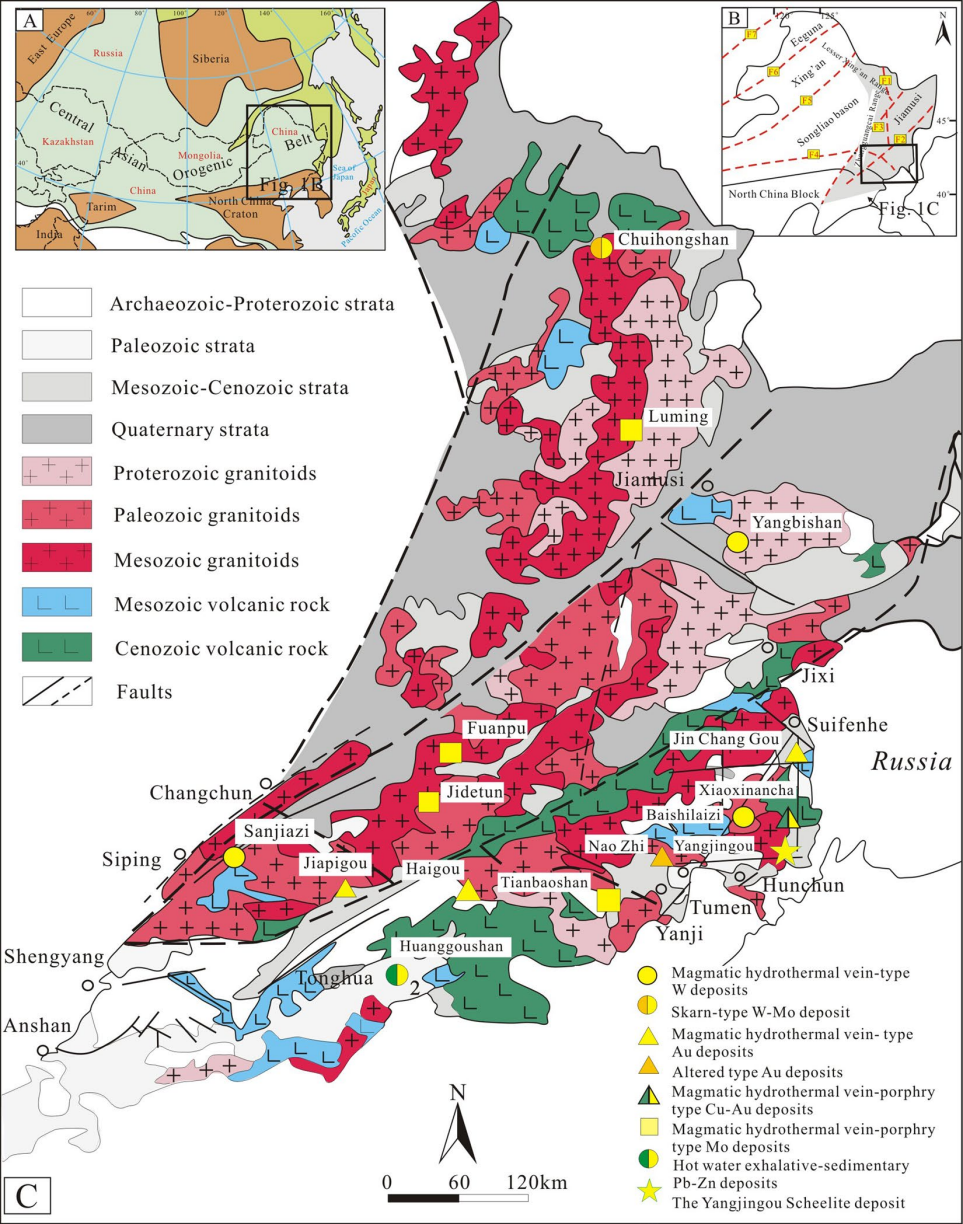
Geological map showing the main units in the Jilin and Heilongjiang metallogenic belt as well as ore deposit locations in the Yanbian area. (**A**) Location of the Central Asian Orogenic Belt and NE China; (**B**) Location of the Jilin and Heilongjiang metallogenic belt; (**C**) Location of the major tectonic units in the Jilin and Heilongjiang metallogenic belt (After Wu^55^; https://www.researchgate.net/publication/229317465_Geochronology_of_the_Phanerozoic_granitoids_in_Northeastern_China).

The Yangjingou scheelite is the largest identified scheelite deposit in the Yanbian belt, NE China. It is located between the Cuihongshan and Sanjiazi scheelite deposits in the Heilongjiang-Jilin metallogenic belt, 8 km south of the Xiaoxinancha Au deposit, approximately 25 km east of the Russian border (Fig. 1c). In this deposit, the main economic mineral is scheelite, accompanied by minor Au, Cu, Mo, Fe, and P mineralization. The area is dominated by low-grade metamorphic rocks of the Wudaogou Group, which formed during a regional episode of low-grade epidote to low-grade amphibolite metamorphism. Regional stratigraphic correlations, metamorphic studies, and U–Pb geochronology from metamorphic detrital zircons indicate that sedimentation and regional metamorphism dated 323 ± 23 Ma^10^ and 266–249 Ma, respectively^11^. The Wudaogou Group is subdivided into the lower Madida Formation, the middle Yangjingou Formation, and the upper Xiangfangzi Formation^12^. The rocks from these formations are composed of muscovite, K-feldspar, amphibolite, plagioclase, quartz, and small amounts of cordierite, andalusite, rutile and kaolinite^12,13^.

**Figure 2:**
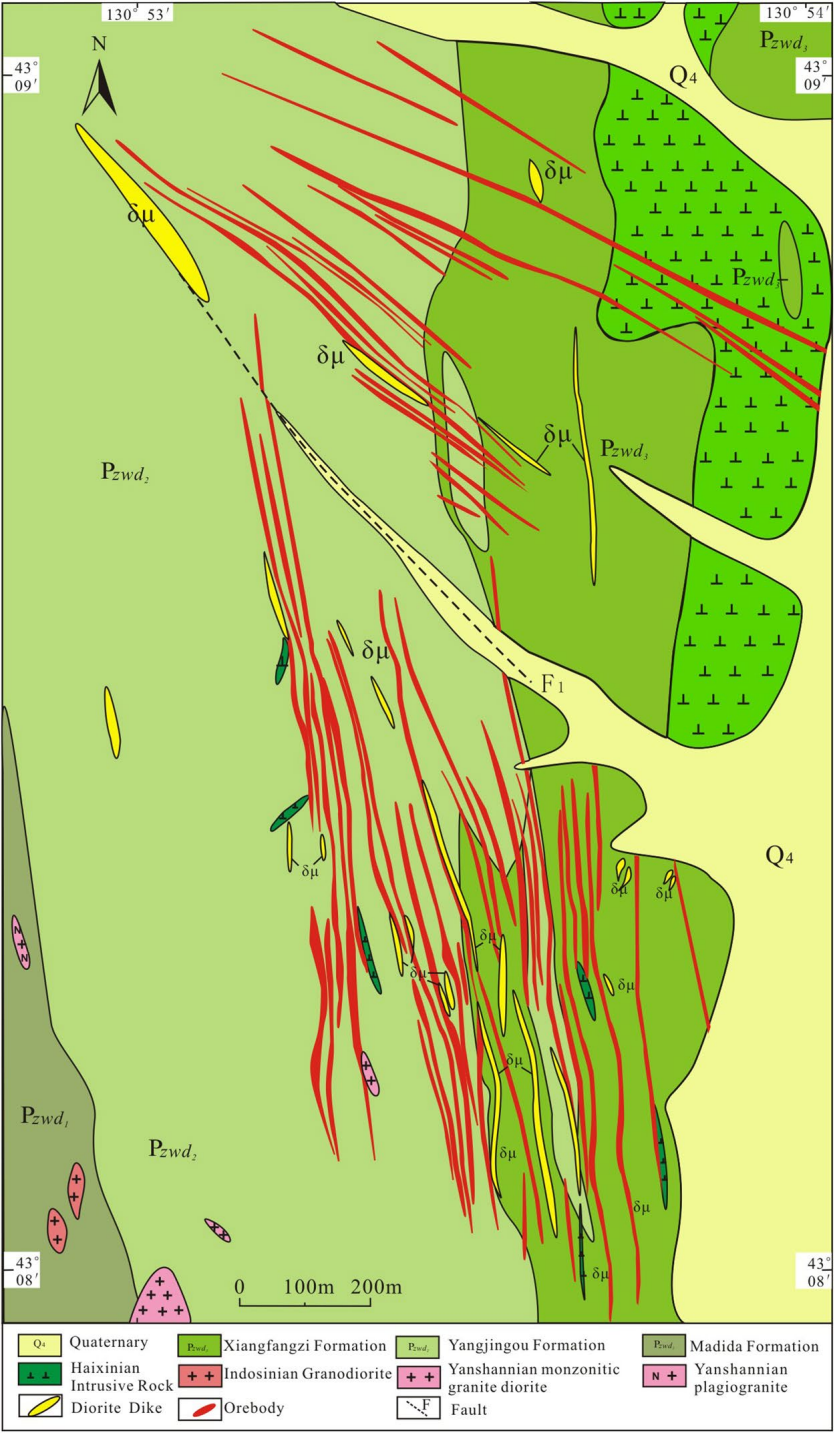
Geological map of the Yangjingou scheelite deposit (After Ren^14,18^; http://www.ysxb.ac.cn/ysxb/ch/reader/create_pdf.aspx?file_no=20101222&flag=1&journal_id=ysxb&year_id=2010).

The Yangjingou scheelite deposit (Fig. 2) is located within a N-S-oriented asymmetrical syncline on the western limb of the Wudaogou synclinorium. The eastern limb dips 45–50°NE, and the western limb dips 55–60°NW (and locally up to 75–80°). The mine is situated in a NW-SE-oriented secondary syncline with an E-W-striking fold axis, crosscut by a N-S- to NW-SE-striking fault. The eastern limb of the synclinorium has been extensively modified by magmatic intrusions, whereas the western limb remains relatively unaltered. The scheelite ore bodies are structurally controlled by 3 secondary N-S-, NW-SE-, and NNW-SSE-striking normal faults. The N-S-striking fault is 2 km in length, dips 60–80°NW and lies parallel to the principle compressional fault in the region. The 0.8–1.0 km NW-NNW-oriented fault, which dips 40–70°SW and lies oblique to the major compressional fault and is extensively mineralized

Several generations of magmatic intrusions, including late Hercynian granodiorite (267 ± 1 Ma)^14^ and early Indosinian Yangjingou granodiorite (249.4 ± 2.7 Ma)^15^ have locally deformed rocks of the Wudaogou Group. Based on the Yangjingou granodiorite chemical signature, a transition from I- to S-type magmatism is observed, interpreted as a result of subduction^16^. Yanshanian intrusives, including monzonitic granodiorite (178.5–197 Ma)^17^ and dioritic porphyry dykes (120.73–157.27 Ma)^17^ were later emplaced within zones of pre-existing structural weakness. Previous studies have revealed a close association between Indosinian magmatism and W mineralization in the Yangjingou deposit^16,18^. Furthermore, Shan^19^ and Hu^17^ suggest that the dikes present in our study area are genetically related to an unexposed granite intrusion.

**Figure 3:**
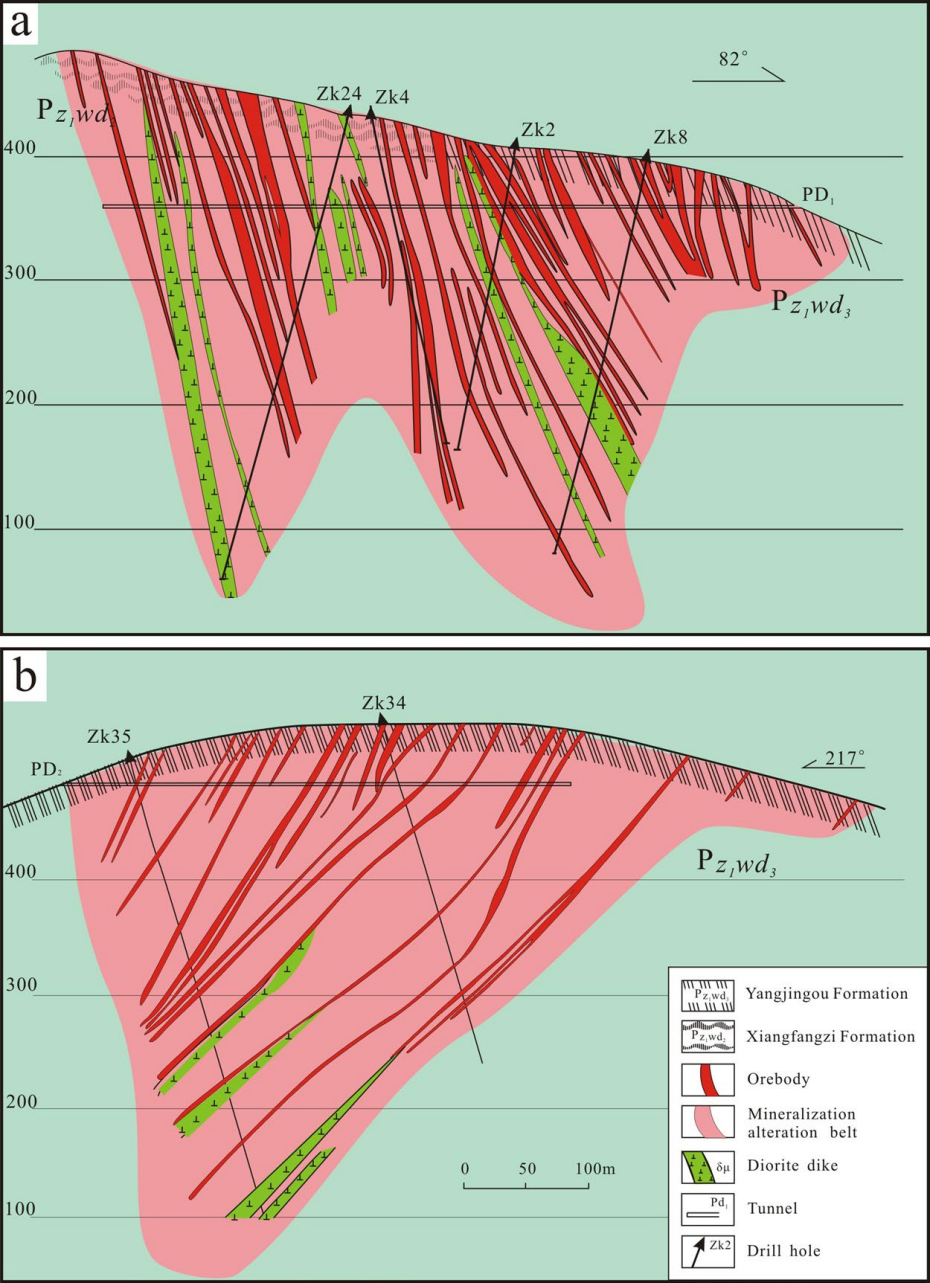
(**a**) Cross section of the southern ore block in the Yangjingou scheelite deposit; (**b**) Cross section of the northern ore block in the Yangjingou scheelite deposit (After Shan^19^; http://kckc.org.cn/ch/reader/create_pdf.aspx?file_no=2010Z106&year_id=2010&quarter_id=Z1&falg=1).

The scheelite ore bodies are mainly hosted in Ca-rich mica schists. The deposit can be subdivided into a southern and a northern ore block, separated by a N-W-trending fault (Fig. 2). In the northern ore block (Fig. 3b), mineralization is structurally controlled by normal faults striking 295–315° and dipping 65–75°. Mineralization occurs in quartz stringers and large quartz veins that have been emplaced along intraformational faults, which partially crosscut the upper Hercynian granodiorite. Individual veins are typically sub-parallel, appear as lenticular or spindle-shaped bodies, exhibit pinching and swelling, and measure approximately 10 cm in width. The ore bodies exhibit a total length of more than 900 m, a width from 60 to 425 m, and a stacking thickness of approximately 95 m.

Mineralization in the southern ore block is structurally controlled by normal faults striking 300–350° and dipping 60–75° (Fig. 3a). The width of individual veins ranges from 2 to 10 cm (locally reaching up to 65 cm). The total length of the ore bodies is approximately 800–1400 m, with a maximum width of 450 m. The scheelite-bearing veins in both the northern and southern ore blocks share similar characteristics in terms of their orientation and geometry.

**Figure 4:**
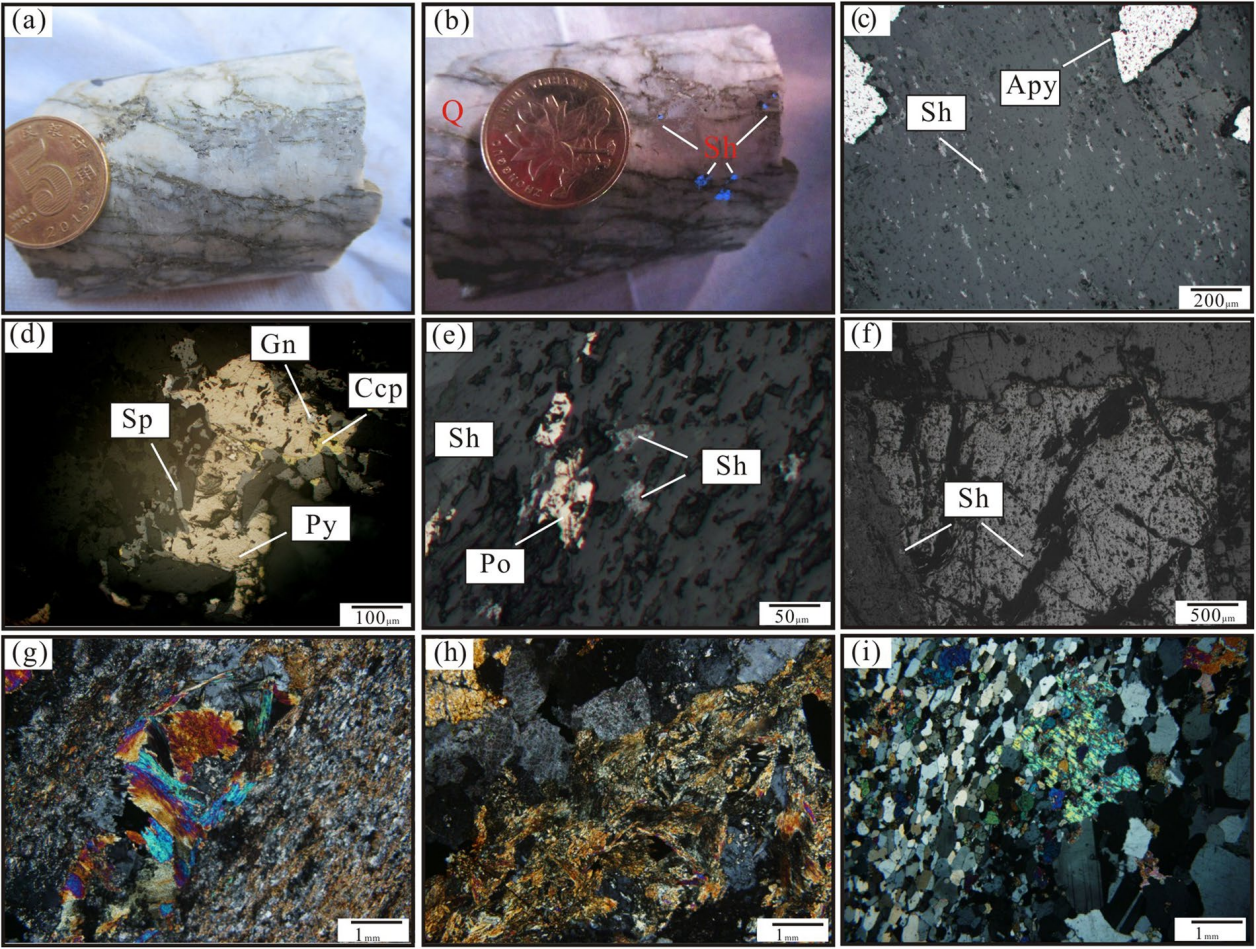
(**a** and **b**) Scheelite paragenetic quartz veins (scheelite showing blue-fluorescence under the ultraviolet irradiation); (**c**) arsenopyrite replaced early scheelite minerals and existed in early disseminated W-mineralizationquartz veins; (**d**) sphalerite and copper pyrites replacing pyrrhotite; (**e**) pyrrhotites replaced early scheelite; (**f**) subhedral scheelite; (**g** and **h**) actinolitization, epidotization; (**i**) muscovite quartz schist.

The average W oxide (WO_3_) grade of the mine is 0.22% to 1.5%, reaching up to 5.25%, with accessory Au (0.49–0.79 g/t), Cu, Mo, Fe, and P^19^. Scheelite mineralization predominantly occurred via micro-vein dissemination and thin vein mineralization (Fig. 4a,b). The ore minerals include scheelite, pyrrhotite, pyrite, arsenopyrite, and sphalerite, while the gangue assemblage is comprised of quartz, muscovite, hornblende, andalusite, epidote, and actinolite. Sulfide minerals typically exhibit euhedral to subhedral crystal habits with granular embayments and metasomatic relict textures. Scheelite grains are subhedral (Fig. 4f) and disseminated in microfractures within quartz veins with a low sulfide content (Fig. 4c). Arsenopyrite has replaced early scheelite minerals and occurs as euhedral-subhedral disseminations in W-mineralized quartz veins (Fig. 4c). Pyrite postdates pyrrhotite and together with chalcopyrite, replaces pyrrhotite. Based on the mineral assemblages, micro-textures and associated alteration types, two stages of mineralization are distinguished: (1) the quartz-scheelite stage (oxide stage) and (2) the later pyrrhotite-arsenopyrite-pyrite stage (sulfide stage), with the quartz-scheelite stage being the major metallogenic stage. The alteration of the ore bodies mainly includes sericitization, actinolitization, albitization, carbonatation, and epidotization (Fig. 4h,I,k).

## Analysis and Methods

### Fluid inclusions

Twenty-six highly transparent 100–300-μm doubly polished thick quartz plates of paragenetic scheelite quartz veins from the southern and northern ore blocks of the Yangjingou deposit were analysed. Fluid petrography and microthermometry analyses were performed at the Geological Fluid Analysis Center of Jilin University, Changchun, China. The microthermometric analysis of fluid inclusions was conducted with a Linkam THMSG 600 stage mounted on a Carl Zeiss Axiolab microscope (10 × 50) capable of temperature measurements from −196 to 600 °C. The accuracy between 31 °C and −100 °C is better than ±0.2 °C and better than 5 °C at >300 °C. The heating/freezing rate is generally 0.2–1 °C/min but is reduced to 0.1 °C/min close to the phase transformation point. The FLUIDS^20,21^ software package used for the calculation of the isochors and the phase transition temperature of N_2_-CO_2_ were plotted in the diagram of Thiery^22^ and Van den Kerkhof & Thiery^23^ in order to determine *v*X properties of the fluid.

### Raman microspectroscopy analysis

**Figure 5:**
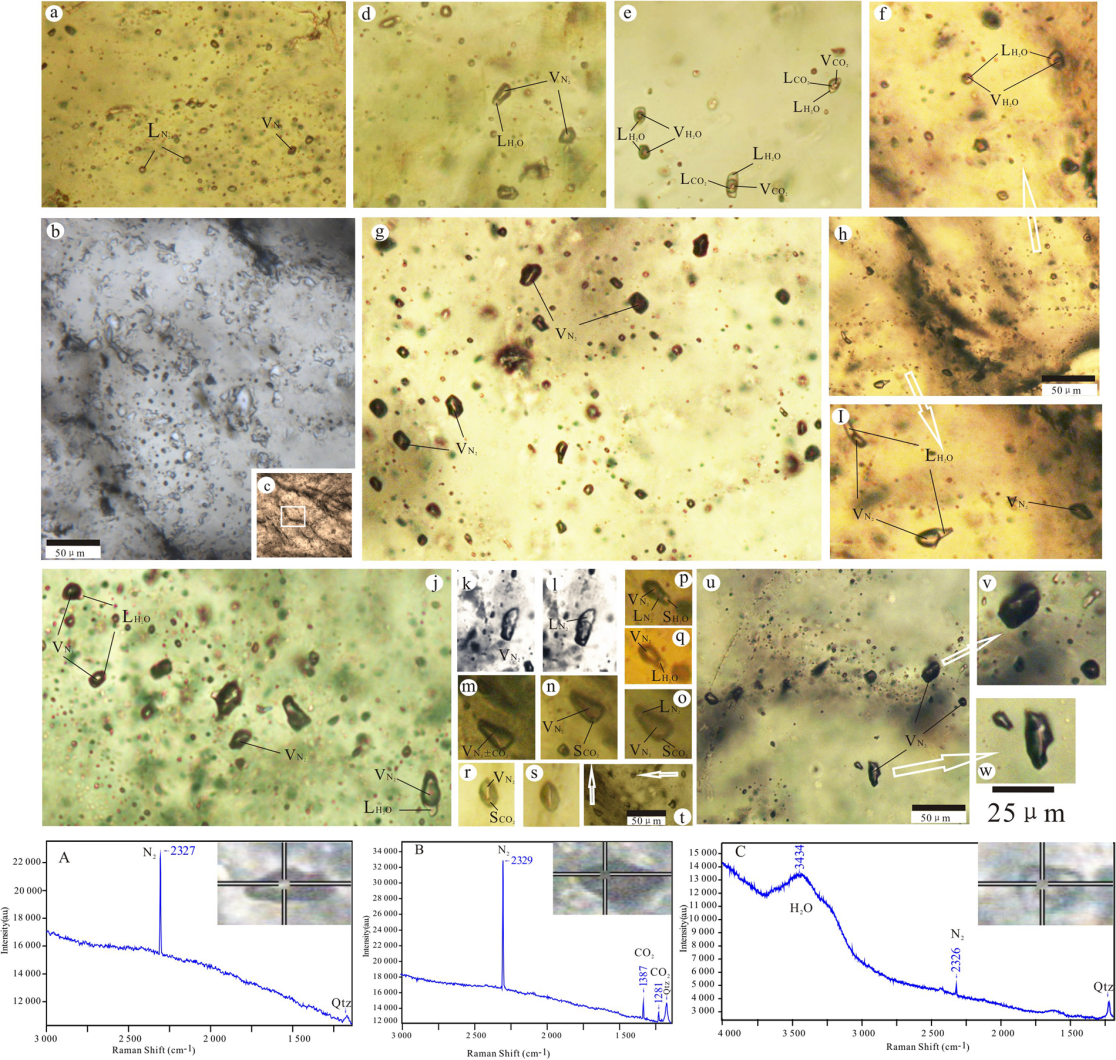
Photomicrographs of representative fluid inclusion types at room temperature in the quartz–scheelite stage. Type I: pure N_2_ fluid inclusions (**a**–**c**,**g**,**u**); Type II: CO_2_–N_2_ inclusions (**o**,**m**,**n**), occasionally solid CO_2_ phase below −100 °C (**n**,**r**) and three phases under the −160 °C (**o**); Type IIIa: inclusions containing 10%–30% gas (**e**,**f**); Type IIIb, inclusions in which the proportion of the gaseous phase ranges from 70% to 95% (**d**,**j,i**), and the liquid phase of N_2_ partially exists at temperatures below −150 °C (**p**). One phase (**k**) at room temperature and two phases below −160 °C (**l**); Type IV: NaCl–H_2_O–CO_2_ fluid inclusions (**e**). Representative Raman spectra of fluid inclusions related to the mineralizing fluid of the Yangjingou scheelite. Panel A shows that the vapour bubbles are mainly composed of N_2_; panel B shows that the bubbles contain mainly N_2_ and some CO_2_; and panel C shows that the fluid inclusions are mainly composed of N_2_ and H_2_O.

Raman analysis was performed at the Geological Fluid Analysis Center of Jilin University. The Raman instrument is a Renishaw RM-1000 and was mounted on an Olympus BX40 microscope. A 532 nm green diode-pumped solid state laser was used as an excitation source. The spectrum counting time was 30–60 s, the spectral resolution 1–2 cm^−1^, and the laser beam diameter 1–2 μm. A detailed description of the measurement procedure was provided by Wopenka^24^. The measurements reveal that the major gaseous fluid inclusion components are N_2_ and CO_2_. A large proportion of the inclusions show only a single strong band at 2328.2–2328.3 cm^−1^, indicating pure N_2_, which is the first time pure N_2_ inclusions have been observed in the W deposit (Fig. 5). Some of the inclusions feature peaks at 3399–3500 cm^−1^, indicating the presence of water. The CO_2_ component corresponds to the Fermi diad at 2328–2330 and 1280–1386 cm^−1^.

### Stable isotope analysis

The oxygen and hydrogen isotopic compositions of primary fluid inclusions in the main stage (oxide stage) of the disseminated scheelite quartz veins were analysed at the analytical laboratory of Beijing Research Institute of Uranium Geology (BRIUG), China, using a MAT253-type mass spectrometer. Grains (40–60 nm) were handpicked to ensure the absence of mineral impurities. Oxygen was liberated from quartz for isotopic analyses via quantitative reaction with BrF_5_ using a CO_2_ laser as a heat source. H_2_O was first released by heating the samples to temperatures above 550 °C then reacted with zinc at 800 °C to produce hydrogen. The results are reported in per mil (‰) relative to the Vienna Standard Mean Ocean Water (VSMOW) standard, with precisions of ±2‰ for δD and ±0.2‰ for δ^18^O at the 1σ level. δ^18^O_quartz_ was measured in order to calculate the δ^18^O_H2O_ values of hydrothermal fluids using the following equation:

$$1000{\mathrm{ln}{\rm{\alpha }}}_{\text{quartz}-\text{water}}=3.38\times {10}^{6}\cdot {{\rm{T}}}^{-2}-3.40,$$

where T represents the homogenization temperature of the fluid inclusions^25^.

Seven quartz samples from scheelite quartz veins were selected for N isotopic analysis. In order to exclude atmospheric interference, samples were degassed for 15 minutes under vacuum (<10^−3^ Pa). N_2_ was then directly released from fluid inclusions by heating the quartz samples in a high-purity quartz cell to temperatures above 600 °C. The released N_2_ was then separated using a chromatographic column cold trap^26^. Nitrogen isotopic compositions of the samples are reported using δ notation, where δ^15^N_sample_ (‰) = [(^15^N/^14^N) _sample_/(^15^N/^14^N) _standard_^−1^] ×1000. The results are reported in per mil (‰) relative to atmospheric N_2_^27^, and the precision is ±1‰ at the 1σ level.

## Results

### Fluid inclusions

The criteria by Roedder^28^ for defining primary, secondary and pesudosecondary inclusions were applied. However, microtheromology measurements were conducted on primary fluid inclusions, because only primary inclusions represent W-mineralized fluid. Primary inclusions occur in isolation or as random clusters within intragranular quartz crystals^28^. Our study focuses on the main W-mineralizing stage, i.e., the scheelite-quartz stage. Based on composition, phase proportion at room temperature (21 °C) and phase transition during total homogenization, four types of fluid inclusions have been identified in quartz crystals from the scheelite paragenetic quartz veins: pure N_2_ fluid inclusions (Type I), CO_2_-N_2_ fluid inclusions (Type II), CO_2_ ± N_2_-NCl-H_2_O inclusions (Type III) and aqueous two-phase VL inclusions (Type IV; Table 1).

**Table 1:** Overview of the four fluid inclusion types observed in mineralizing quartz veins in the Yangjingou scheelite deposit.

W-quartz vein	N_2_	N_2_−CO_2_	CO_2_ ± N_2_+ H_2_O + NaCl	H_2_O + NaCl ± N_2_
YJ-1	N_2_ >90 mole%	N_2_: 46–76 mole%	Gas phase: N_2_: 8–24 mole%	Gas phase: N_2_
		CO_2_: 24–54 mole% v	CO_2_: 76–92 mole%	a: Size 4–16 μm
	V_m_: 35~60 cm^3^/mole	30– 48 cm^3^/mole	Size: 6–20 μm	Fill 10%–20% Vol. Fracs
	Size: 8~10 μm	Size: 8–25 μm	Fill: CO_2_/L_H2O_ 50–70% L_CO2_/V_CO2_: 70–85% 40~60	T_h_250 °C–400 °C
	Fill 100% Vol. Fracs	Fill 100% Vol. Fracs	T_h_: 283– 417 °C	Sal 5.7–9.6 wt% NaCl eq
	T_h_ (L)_N2_: −153 °C~−168 °C	T_hN2_: −147.5 °C~−163.2 °C	T_mCO2_: −62~−58.1 °C	b: Fill 70–90% Vol. Fracs
	T_h_ (V)_N2_: ~−150.3 °C	T_hCO2_: −46 °C~−86 °C	T_CO2clathra_: 5.9–8.2 °C	T_h_ 315–410 °C
		T_mCO2_: −60.9 °C~−60.7 °C	T_hCO2_: 9.8 °C~ 17.5 °C	
		V_m_: 30–65 cm^3^/mole	Sal: 3.57–7.58 wt% NaCl eq	
YJ-3	N_2_ >90 mole%	N_2_: 42–78 mole% N_2_: 46~76 mole%	Gas phase N_2_: 8~24 mole%	Gas phase N_2_
		CO_2_: 22–57 mole%	CO_2_: 78~92 mole%	a: Size 4~14 μm
	V_m_: 36~59 cm^3^/mole	V_m_: 30–47 cm^3^/mole	Size: 8–18 μm	Fill: 10–15% Vol. Fracs
	Size: 4~28 μm	Size: 9–24 μm	Fill CO2/L_H2O_ 55–75% L_CO2_/V_CO2_ 70–85% 40~60	T_h_: 271–381 °C
	Fill 100% Vol. Fracs	Fill: 100% Vol. Fracs	T_h_: 290- 423 °C	Sal 5.7- 9.47 wt% NaCl eq
	T_hN2_: −153.4 °C~−168 °C	T_h N2_: −147.7 °C~−162.3 °C	T_mCO2_: −62.1~−59.5 °C	
	T_h_ (V)_N2_: ~−150.4 °C	T_hCO2_:−46 °C~−86 °C	T_CO2clathrat_: 5.6−7.5 °C	b: Fill 75- 95%
		T_mCO_: −60.8 °C~−60.7 °C	T_hCO2_: 10.6 °C- 18.4 °C	T_h_: 335- 396 °C
		V_m_: 30–60 cm^3^/mole	Sal: 4.87- 8.13 wt% NaCl eq	
YJ-4	N_2_ >88 mole%	N_2_: 46–79 mole% N_2_: 46~76 mole%	Gas phase N_2_: 8~24 mole%	Gas phase N_2_
		CO_2_: 21–54 mole%	CO_2_: 78- 89 mole%	a: Size 6–12 μm
	V_m_: 34~59 cm^3^/mole	V_m_: 31- 45 cm^3^/mole	Size: 6- 20μm	Fill: 10–30% Vol. Fracs
	Size: 8~26 μm	Size: 8–26 μm	Fill CO_2_/L_H2O_ 50- 60% L_CO2_/V_CO2_: 70- 90% 40~60	T_h_: 285–378 °C
	Fill 100% Vol. Fracs	Fill 100% Vol. Fracs	T_h_: 290 °C- 422 °C	Sal: 5.85- 9.21 wt% NaCl eq
	T_hN2_: −151.2 °C~−167.5 °C	T_hN2_: −148.4 °C~−162 °C	T_mCO2_: −65 °C~−60.8 °C	b: Fill 70- 95% Vol. Fracs
	T_h_(V)_N2_: ~−150.3 °C	T_hCO2_: −46 °C~−86 °C	T_CO2clathrat_: 5.3 °C- 7.5 °C	T_h_: 339- 410 °C
		T_mCO2_: −60.8 °C~−60.7 °C	T_hCO2_: 10.8 °C- 20.5 °C	
		V_m_.: 30–62 cm^3^/mole	Sal: 4.87%- 8.45 wt% NaCl eq	
Transition	L+G->L(G)	H-type: S+L+V->S+L(G)-> S+L+G->L+G->L(G) S-type: S+L+G->S+L(G)->L(G)	S->S+L->S+L+G->L+G>L	S->S+L->S+L+G->L+G->L(G)

**Figure 6:**
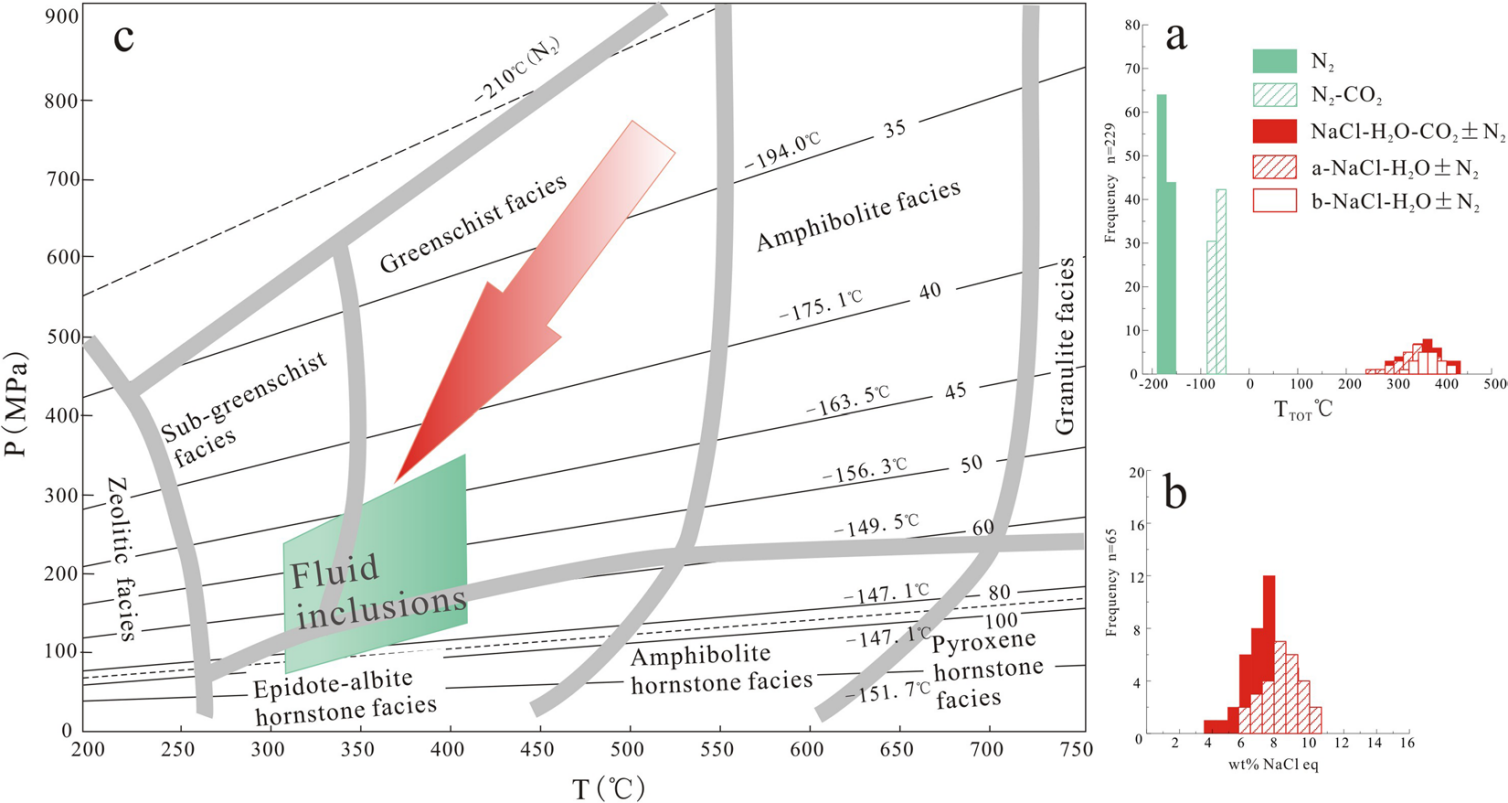
(**a**) The homogenization temperature of the fluid inclusions (Type I, II, III and IV). (**b**) Salinity of type IIIa and IV fluid inclusions. (**c**) The P–T conditions of the fluid inclusions in the scheelite quartz veins in the Yangjingou scheelite deposit (molar volums between 30 and 500 cm^3^/mole) (After Kerkhof amd Thiéry^23^ and Winter^52^). Numbers denote molar volumes in cm^3^/mole; numbers in brackets are the homogenization temperature of N_2_.

The Type I (pure N_2_) fluid inclusions (Fig. 5a,b,c,g,u) occur as a single phase at room temperature and are 6–30 μm in size. The inclusions are mostly oval in shape or exhibit a negative crystal shape and are randomly distributed in quartz grains. Type I inclusions are identified as N_2_ homogenizes to liquid or gas (L + V- > L(G)) and are closely related to scheelite deposits, but have been rarely reported. At low temperatures, bubbles nucleate at −162 °C~−182 °C. The final homogenization of the liquid phase T_h_(L) occurs between −151 °C~−168 °C, although a few Type I fluid inclusions homogenize to the gas phase at −150.3 °C (Fig. 6a,c). Raman analysis of the Type I inclusions yields compositions of >88 mol% N_2_, with minor amounts of CO_2_ exhibiting molar volumes of 42–150 cm^3^/mol^22,23^ (Fig. 6c). No other gases were identified.

**Figure 7:**
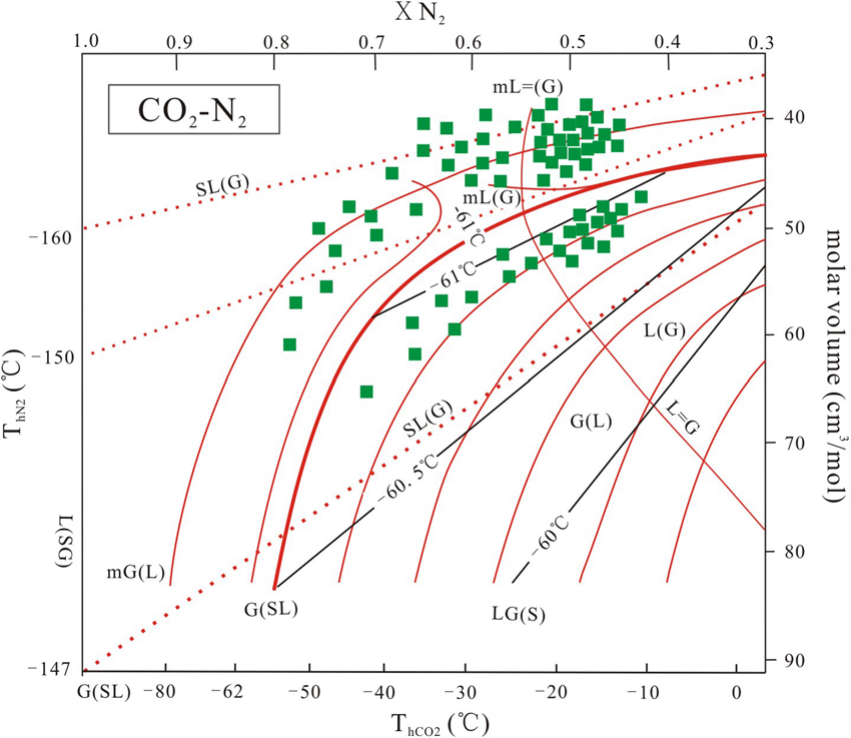
vX diagrams calculated for the CO_2_-N_2_ system of the Yangjingou deposit showing low molar volumes (v < 100 cm^3^/mole). The thick red line G (SL) defines the difference between S-and H-typeII inclusions. Inclusions plotting below the L = G homogenize to gas, above the critical curve to liquid. Thin read solid line = homogenization (T_hCO2_). Dashed dotted red line = partial homogenization (T_hN2_). Thin black line = initial melting of CO_2_ (T_mCO__2_) (After Kerkhof and Thiéry^23^; Thiéry *et al*.^22^).

The Type II (CO_2_–N_2_) inclusions (Fig. 5m,s) are 5–25 μm in size, and most are between 8–15 μm. These inclusions exhibit euhedral and negative crystal habits and are randomly distributed in the quartz grains. During cooling, the inclusions show bubble and solid CO_2_ nucleation. The phase transitions on warming follow one of two sequences: S+L+G->S+L(G)>S+L+G->L+G->L(G) (H-type, i.e. T_h_ > T_m_), showing homogenization as the final phase transition or S+L+G->S+L(G)->L(G) (S-type, i.e. T_h_ < T_m_) showing sublimation as the final phase transition^29,30^. Bubbles of nitrogen nucleate between −178 and −159 °C (Fig. 5o); solid CO_2_ is infrequently observed below −100 °C (Fig. 5n,r). In S-type inclusions the partial homogenization (T_hN2_) to a liquid (gas) of is between −163.2 and −147.5 °C. Critical homogenization can be observed around −147 °C and the total homogenization (T_hCO2_) is characterized by sublimation to liquid (gas) phase from −86~−69 °C. The T_hN2_ of H-type inclusions to the liquid (gas) phase coincided with critical homogenization and occurred between −149 and −148 °C. The total homogenization (T_hCO2_) to liquid (gas) varied from −60.8 to −45.6 °C with the T_mCO2_ between −60.9 and −60.7 °C. Raman spectroscopy shows that the inclusions are composed of 42–79 mol% N_2_, and 21–58 mol% CO_2_ with molar volumes between 30 and 65 cm^3^/mole, in agreement with microthermometry results (Figs 6a and 7).

The Type III (NaCl–H_2_O) inclusions contain liquid and gas at room temperature and have been subdivided into Type IIIa and Type IIIb populations. Type IIIa inclusions contain gas volume fractions of <50%, where most are between 10 and 30% (Fig. 5f). N_2_ less than 10% were infrequently detected in these inclusions. Fluid inclusions are generally 4–16 μm in size, clustering between approximately 6 and 10 μm and appear as elongated and spindle shapes, which are randomly distributed in quartz grain. These inclusions yield Tm_ice_ of −6.7 °C to −3.5 °C and homogenize to liquid between 250 and 400 °C, predominantly between 300 and 375 °C (Fig. 6a). Therefore, the salinity of these inclusions is 6–10 equiv. wt% NaCl^20^ (Fig. 6b). In Type IIIb inclusions, the gas volume fractions are >50%, where most are between 70–95% (Fig. 5d,j,i). The total homogenization temperature to the gas phase occurs between 314 and 409 °C, predominantly between 334 and 381 °C (Fig. 6a). The liquid phase of N_2_ can be locally observed at temperatures below −150 °C (Fig. 5p).

The Type IV (NaCl–H_2_O–CO_2_ ± N_2_) inclusions contain CO_2_ gas, CO_2_ liquid, and a saline solution at room temperature (Fig. 5e). The volume fraction of CO_2_ bubbles in the inclusions exceed 40%, with the majority of the inclusions featuring volume fractions of 50–80%. The gas phase comprises 75–90% of this volume fraction. These primary inclusions show euhedral and irregular polygonal shapes. They occur in randomly distributed clusters in quartz grains and individual inclusions are small, measuring 4–20 μm. Solid CO_2_ melts at −60.8~−58.1 °C, indicate the presence of minor N_2_. CO_2_ clathrate melting occurs at 5.3–8.1 °C and the salinity of the inclusions ranges from 4 to 9 equiv. wt% NaCl (Fig. 6b). Partial homogenization of CO_2_ to liquid occurs at 9.8–20.5 °C, and total homogenization to liquid occurs at 283–423 °C, predominantly at 320–402 °C (Fig. 6a).

### Stable isotopes

**Figure 8:**
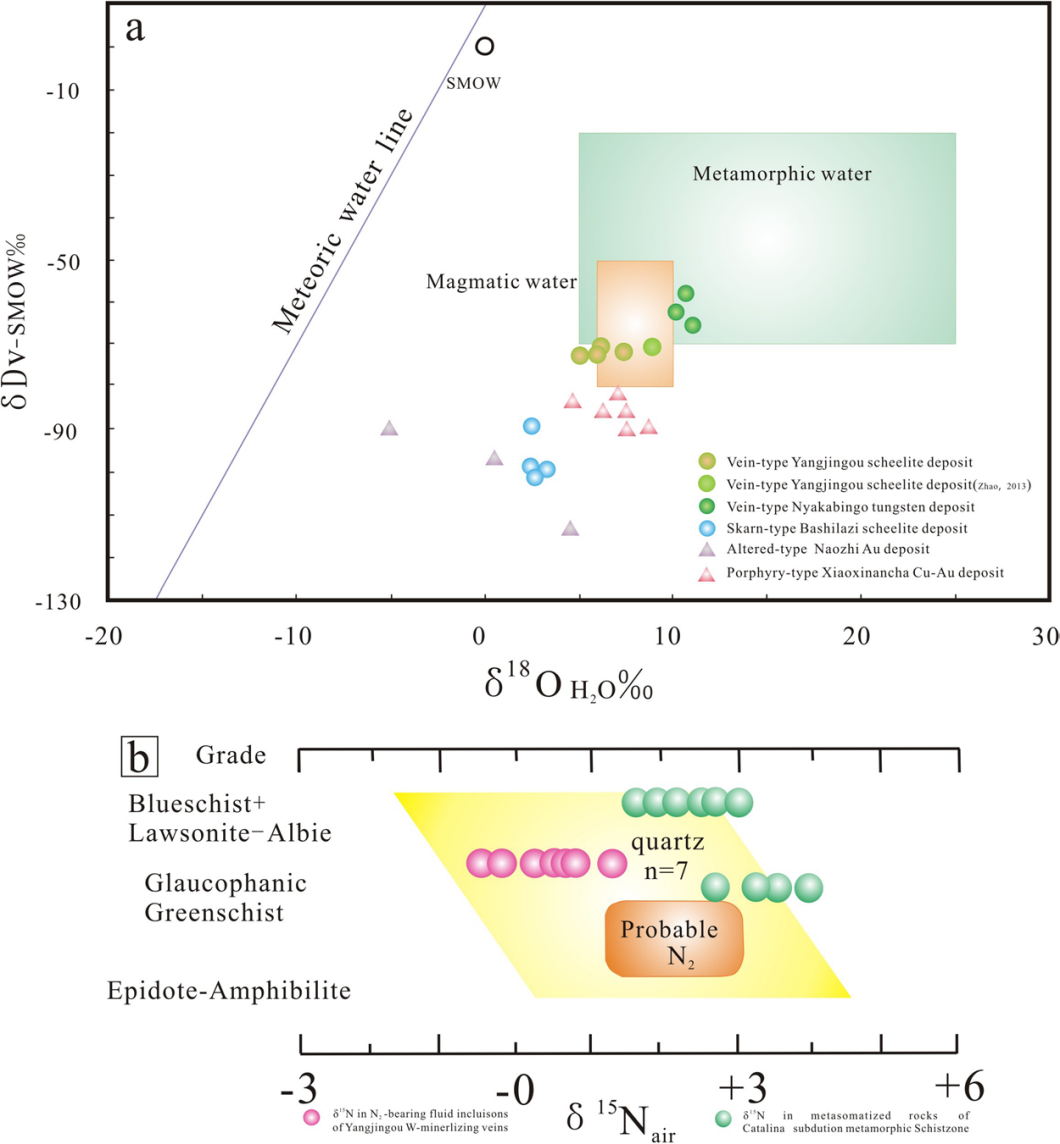
(**a**) Plot of δD values versus δ^18^O values for the ore-forming fluids related to the Yangjingou scheelite deposit (modified after Taylor^56^). The data are from the following sources: Naozhi Au deposit (Huang *et al*.^41^), Xiaoxinancha Au deposit (Wang^42^), Baishilazi W deposit (Zhao^16^) in Yanbian area and Nyakabingo W deposit, central Africa (Dewaele *et al*.^7^). (**b**) Nitrogen isotopic compositions of the fluid inclusions in the Yangjingou scheelite deposit and (After Bebout *et al*.^27^).

The δD and δ^18^O values of the W-mineralized quartz veins exhibit relatively narrow ranges (−74.9~−77‰ and 9.6~12‰, respectively; Table 2). Wood and Samson^31^ demonstrated that scheelite precipitation in W deposits hosted by siliceous rocks occurs at temperatures of 200–500 °C. Consequently, the mode homogenization temperature of 375 °C in the Type IIIb fluid inclusions was chosen to calculate the δ^18^O value of the water in the ore-forming fluid. In the δ^18^O–δD diagram, the calculated δ^18^O_H2O_ values (4.96−7.36‰; Fig. 8a) fall within or close to the fields of metamorphic water and primary magmatic water. The δ^15^N values of the N_2_ extracted from the fluid inclusions vary from −0.5‰ to 1.4‰ (Table 2; Fig. 8b). In the δ^15^N graph, all the samples from the W-mineralized quartz veins fall in the range characterized by sub-greenschist-facies metamorphism.

**Table 2:** Hydrogen and oxygen isotopic compositions of the main stage quartz in the Yangjingou scheelite deposit.

Mineral	Mineral	Sample	δD (‰)	δ^18^O (‰)	δ^18^O_H2O_ (‰)	δ^15^N (‰)	
		Q11	−75	13.5	8.20		Zhang *et al*.^15^
Yangjingou Scheelie deposit	W-mineralizing quartz vein	Y-1	−77	9.6	4.30	0.7	This study
		Y-2	−74.9	10.8	5.50	0.4	
		Y-3	−76.1	12	6.70	−0.2	
		Y-4	−76.7	10.6	5.30	−0.5	
		YJ-10				0.6	
		YJ-7				1.4	
		YJ-8				0.8	
Naozhi Au deposit	Au-minerlizing quartz veins	NB124-1	−123.8	4.598	−1.40		Huang *et al*.^41^
		NB123	−97.3	7.525	0.53		
		NB601	−113.05	10.562	4.53		
		NB119	−90.62	4.583	−5.01		
Baishilazi W-deposit	W-minerlizing quartz veins	BS-4	−100.6	13.1	3.14		Zhao^16^
		BS3-1	−102.4	12.6	2.64		
		BS-2	−100.4	12.4	2.44		
		BS-6	−90.8	12.4	2.44		
Nyakabingo W-deposit	W-minerlizing quartz veins	Ny09FD02	−57		15.4		S. Dewaele^7^
		Ny09FD08	−64		15.6		
		Ny09FD11	−61		14.9

## Discussion

### Nitrogen in ore-forming fluids

The presence of N in W-bearing mineralizing fluids has previously been reported^7,8,32^; however, fluids with >50 mol% N_2_ are rare. Lin^33^ assumed that the N_2_-rich fluid inclusions identified at the Dongchuan Cu deposit resulted from either mantle devolatization during the break-up of Rodinia or the trapping of gas derived from decomposing organic materials. In contrast, the Yangjingou deposit formed in a compressional environment during the closure of the Paleo-Asian Ocean. There is no evidence for mantle degassing during regional orogenic activity in this area. Therefore, the presence of N_2_ in the deposit may be associated with plate collision, ocean closure, regional Barrovian metamorphism, and eclogite-facies metapelites^34,35^.

Under alkaline conditions, W is transported in the form of H_2_WO_4_, M_2_WO_4_ (M = Na, K), and WO_4_^−2^ complexes in W-mineralizing fluid. The presence of NH_4_^+^ in the solution stabilizes the W polyacid, thereby increasing its compatibility in migrating fluids^36^. The stability of NH_4_^+^ (substituting for K^+^) widely depends on the metamorphic and oxygen fugacity conditions^27,37^. Nevertheless, under metamorphic conditions, NH_4_^+^-bearing minerals may release N_2_ (350–600 °C) or NH_3_ (650–700 °C) at higher oxygen fugacity and concentrate in inclusions. The following equation illustrates the production of N_2_ under oxidizing conditions^38^:

1 $${{{\rm{NH}}}_{4}}^{+}+2{{\rm{O}}}_{2}={{\rm{N}}}_{2}+2{{\rm{H}}}_{2}{\rm{O}}$$

The main cause for scheelite precipitation is an increase in the activity of Ca^2+^, which results in the chemical equilibration of the mineralizing fluid with the host rock and has been recorded at a range of pressures and temperatures^32^. The addition of the non-polar volatile N_2_ via water–rock interactions in mica may decrease scheelite solubility in common metamorphic assemblages. N_2_ has been demonstrated to dominate changes in the distribution of W aqueous species and increase coefficient activity^8^. In other words, an increase in the N_2_ content in the mineralizing fluid may promote scheelite precipitation.

### N_2_-rich fluids in the Yangjingou scheelite deposit

#### Nature of the ore-forming fluids

The ore must have precipitated from H_2_O-CO_2_-N_2_-NaCl-bearing fluids with low to moderate salinity at moderate to high temperatures. The only volatile components in this fluid are CO_2_ and N_2_; hydrocarbons (i.e., CH_4_) have not been detected. From the *v*x plot (Figs 6, 7c) we can observe that the fluid inclusions richer in CO_2_ (30–65 cm^3^/mol) have lower molar volumes than those richer in N_2_(42-150 cm^3^/mol). This has been observed in many metamorphic areas^3,29^ indicating that CO_2_ and N_2_ may not have the same origin^38^. The homogenization temperatures of N_2_ (T_hN2_) and H_2_O ± N_2_-NaCl (T_hH2O_) constrain the pressure-temperature (P–T) conditions of trapping to 315–410 °C at 80–350 MPa (Fig. 6c), suggesting that the W mineralization occurred during late stage greenschist-facies metamorphism at temperature of ~300–600 °C^39^.

#### Stable isotopes

The stable isotopic composition of a vein provides direct information regarding the transport processes during vein formation^25^. Several sources have been suggested for mineralizing fluids in hydrothermal systems. These include magmatic origin, in which fluids evolve in relatively closed systems. Such mineralizing fluids are only observed in association with vein-type wolframite deposits^40^. Several models that result in mineral precipitation from magmatic fluids of mixed origin are as follows (Fig. 8a): 1) a dominantly magmatic fluid mixing with meteoric waters (e.g., the Xiaoxinancha porphyry Cu–Au deposit, Naozhi alteration Au deposit and Bashilazi skarn-type scheelite deposits)^16,41,42^ and 2) the interaction of magmatic fluids with NH_4_^+^-rich host rocks, and/or mixing with metamorphic fluids in equilibrium with metasedimentary lithologies (e.g., the vein-type Nyakabingo W deposit)^7^.

Low δ^18^O and δD of the Yangjingou scheelite deposit indicate that the magmatic component of the fluid played a more dominant role in this deposit compared to the Nyakabingo deposit, in which the mineralizing fluid was metamorphic (Fig. 8a). Earlier C and S isotopic studies yielded δ^13^C_V-PDB_ values of −3.4‰ to −6.5‰ for CO_2_ and δ^34^S_V-CDT_ values of −0.4‰ to 4.7‰ for pyrrhotite, pyrite, and arsenopyrite in scheelite-mineralized veins^15^, consistent with mantle values (δ^13^C_V-PDB_ = −5‰ and δ^34^S_V-CDT_ = ±5‰), demonstrating that the fluid was derived from the mantle. However, compared with magmatic hydrothermal deposits in this area (Fig. 8a), the Yangjingou scheelite deposit shows different δD values which may be interpreted in one of two ways: 1) the original mineralizing magmatic hydrothermal fluid interacted with NH_4_^+^-rich metamorphic minerals and/or mixed with metamorphic fluids during host rock equilibration or 2) the mineralizing fluid of magmatic origin, mixed with low-δD meteoric waters. Because W-mineralized in the presence of Ca-mica schist host rock coupled with N_2_-rich fluid inclusions in the Yangjingou deposit, the first explanation is preferred.

The N isotopic system may potentially provide a detailed record of fluid-rock interaction characteristics and other mixing processes in the crust and mantle^37^. N_2_ has been shown to be released during metamorphism and the breakdown of NH_4_^+^-bearing minerals, such as biotite, cordierite, and white mica^43^. During metamorphism, isotopically “light” N is preferentially fractionated into metamorphic fluids^39,44–46^, and the δ^15^N values of fluid inclusions in quartz veins in low-grade metamorphic rocks yield values ranging from −3 to 5‰^31^. Similarly, the W-mineralized quartz veins in the Yangjingou deposit feature low δ^15^N values (Fig. 8b), which may be attributed to fluid–rock interaction and the continuous liberation of N_2_ in a metamorphic environment. The “light” N is preferentially fractioned into the fluid and is then trapped in the fluid inclusions.

#### The origin of the Yangjingou N_2_-rich fluid

The Wudaogou Group host rock was deposited at 323 ± 23 Ma^10^. From 269-228 Ma, the tectonic regime of the region was dominated by the subduction of the Paleo-Asian oceanic plate beneath the North China plate^47^. Regional greenschist- to epidote-amphibolite-facies metamorphism occurred between 269 ± 4 Ma and 249 ± 4 Ma^13^. The age of muscovite crystallization in the W-mineralized quartz veins is 230.79 ± 1.19 Ma^16^, indicating that W mineralization occurred in the earliest Late Triassic and during late-stage Wudaogou Group metamorphism. Therefore, the fluid system containing N_2_ ± CO_2_–H_2_O–NaCl was intimately associated with complex multiphase tectonics and magmatic activity.

There are three mechanisms by which N_2_ ± CO_2_ may have entered the mineralizing system: 1) trapping of a volatile phase separated from its parent H_2_O–NaCl–CO_2_–N_2_-rich fluid, 2) mixing of a magmatic–hydrothermal CO_2_-H_2_O-rich fluid with N_2_ released via the thermal decomposition of NH_4_^+^-bearing minerals, or 3) CO_2_–N_2_ degassing of the mantle.

The stable isotope data in this study indicate that the mineralizing fluid was of a mixed magmatic and metamorphic origin. Magmatic melt inclusion studies have shown that CO_2_ is released during magmatic differentiation^48,49^, producing a distinct δ^13^C signature^17^. Meanwhile, the Wudaogou Group contributed Ca^2+^ to the system, facilitating the precipitation of Ca(WO)_4_. Within this metamorphic environment, N occurs as NH_4_^+^, which substitutes for K^+^ in detrital phases such as K-feldspar, authigenic clays and illite. With increasing pressure and temperature, both feldspar and clays participate in low-grade metamorphic reactions, and illite gradually transforms into micas^50^. During this transformation, a large amount of carbon is released in the form of CO_2_ due to decarbonization during progressive metamorphism. Moreover, the reported content of CO_2_ in schist (<0.25%) is notably lower than that in clay and shale (2.5–5%)^36^. Thus, the contribution of organic matter to the mineralizing fluid was negligible. No CH_4_ was identified in the W-mineralizing fluid, which may be due to the high oxygen fugacity^51^. Subsequently, Ca^2+^ from the Ca-rich mica schist country rocks was incorporated into the mineralizing fluid. The P–T conditions of the Yangjingou deposit (315–410 °C at 110–350 MPa) are lower than greenschist-facies P–T conditions (350–550 °C at 150–1100 MPa)^52^, indicating that scheelite mineralization likely occurred in the later stages of Wudaogou Group metamorphism. Additionally, the δ^15^N values of the W-mineralized quartz veins show that water–rock interaction occurred under sub-greenschist-facies conditions. However, minor mantle N_2_ participation cannot be completely ruled out.

Yangjingou scheelite mineralization is related to the intrusion of granodiorites at 249 ± 2.7 Ma^16^, synchronous with Wudaogou Group metamorphism. A/CNK (1.1–1.18) and Na_2_O/K_2_O (2.45–7.06)^16^ major element values are characteristic of alkali series I- to S-type transient magmatism. The volatile component of the melts was influenced by pressure, which affected the volatile solubility in the magma during ascent. With decreasing pressure, gas was liberated from the magma^53^, resulting in an increasing volume of CO_2_. CO_2_ dissolves relatively readily in intermediate-alkaline magmas, leading to more widespread and more rapid emplacement of mineralizing fluids^46^. Under these conditions, the diffusion of the W-rich mineralizing fluid was accelerated along interformational structures, causing the fluid to migrate to and rapidly expand into the host rocks of the Wudaogou Group. Only minor N_2_ (<0.25 mol%)^54^ was identified in fluid inclusions in the Xiaoxinancha Cu-Au porphyry deposit (123.35 ± 0.8 Ma)^42^, which is younger than the deposits in the Wudaogou Group and other stratigraphic units. The high concentration of N_2_ observed in the scheelite deposits cannot simply be attributed to organic-rich sedimentary rocks. Findings from this study demonstrate that N_2_-rich fluids are closely related to the degree of metamorphism and magmatic activity.

**Figure 9:**
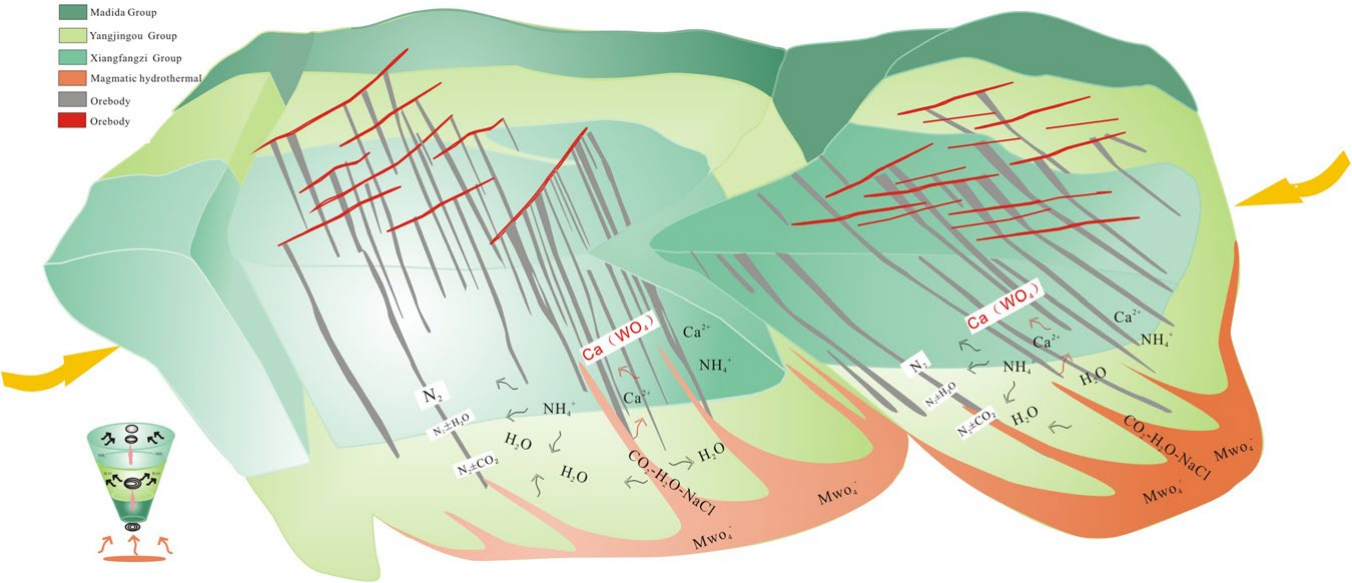
The model of the mineralization of the Yangjingou scheelite deposit under the influence of both magmatism and metamorphism. Magmatic–hydrothermal CO_2_-H_2_O-rich fluid interacted with the host rock and mixed with N_2_–rich fluids during migration, which caused scheelite participation.

Thus, we assume that the N_2_ ± CO_2_ fluid was a product of late-stage Wudaogou Group metamorphism, which caused H_2_O–NaCl–CO_2_-bearing magmatic–hydrothermal fluids containing HWO_4_^−^ and MWO_4_^−^ to migrate through fractures and pre-existing structural weaknesses via fluid circulation, thereby promoting interaction with metamorphic wall rocks (Fig. 9). An initial increase in P-T conditions at ~269 Ma resulted in early low grade metamorphic reactions that transformed feldspar and clays within illite into micas. During this process, NH_4_^+^ substituted for K^+^, resulting in an increase in NH_4_^+^ within the constituent minerals of the Ca-rich mica schists^50^. During late-stage metamorphism (~249 Ma), particularly under greenschist-facies conditions, NH_4_^+^ was released via continuous metamorphism or thermal decomposition (i.e., complete breakdown of host minerals, such as mica)^27,50^. Simultaneously (~249 Ma), the circulation of a magmatic–hydrothermal W-mineralizing fluid under high oxygen fugacity conditions transferred heat (~400 °C) to the surrounding metamorphic rock and broke down micas in the host rock^37,51^. Within an oxidizing environment at this temperature range, large amounts of N_2_ were released from the NH_4_^+^-bearing host rocks and stabilized into fluid^43^. The addition of sufficient amounts of N_2_ to the mineralizing fluid increased activity coefficients and catalysed the saturation of Ca^2+^ and WO_4_^2–^, greatly decreasing the scheelite solubility and accelerating scheelite precipitation. Moreover, saturated fluids, driven by advection, ascended into the surrounding country rock, resulting in alteration (albitization, chloritization, epidotization, and sericitization) and the incorporation of H_2_O into new minerals, leaving N_2_ ± CO_2_ trapped in quartz inclusions. Thus, the N_2_-rich fluid represents W-mineralizing fluid evolution and is indicative of W fluid saturation and scheelite precipitation.

## Conclusions

1. Nearly pure N_2_ (>88 mol%) fluid inclusions have been identified in W-bearing quartz veins in the Yangjingou deposit.
2. Four fluid inclusion populations have been identified in the deposit:

•Type I: N_2_-rich inclusions (N_2_ >88 mol%, T_h_(L) _N2_ = −151 to −168 °C, T_h_(V)_N2_ = −150.3 °C, 42–150 cm^3^/mole).
•Type II: N_2_ ± CO_2_ inclusions (N_2_ = 42–79 mol%, T_hN2_ = −150 to −163 °C, T_hCO2_ = −45.6 to −86.1 °C, CO_2_ = 21–58 mol%, 30–65 cm^3^/mole).
•Type III: NaCl–H_2_O inclusions (a: T_h_ = 250–400 °C, 6–10 equiv. wt% NaCl; b: T_h_ = 315–410 °C).
•Type IV: CO_2_ ± N_2_–NaCl–H_2_O inclusions (CO_2_ = 76–92 mol%, T_h_ = 283–423 °C, 4–9 equiv. wt% NaCl).

3. The fluid inclusions were trapped at 315–410 °C and 80–350 MPa. The NaCl–H_2_O–CO_2_ fluids were magmatic–hydrothermal in origin, and the N_2_ was derived from the breakdown of NH_4_^+^-bearing minerals during alteration.
4. Hydrogen and oxygen isotopic data indicate that the mineralizing fluids were of a mixed magmatic and metamorphic origin, and the nitrogen isotopic data from the W-mineralized quartz veins suggest that water–rock interaction occurred under low greenschist-facies conditions.
5. The N_2_-rich fluid was intimately associated with the scheelite precipitation. During alteration under high oxygen fugacity conditions, N_2_ was released from NH_4_^+^-bearing minerals. The N_2_-rich fluid represents the W-mineralizing fluid evolution, following W saturation and scheelite precipitation. Therefore, areas that have experienced regional metamorphism (orogenesis) and exhibit similar geologic histories to the Yanbian area in NE China are likely candidates for scheelite precipitation as observed in the Yangjingou deposit. In this study, we demonstrate that scheelite precipitation can be genetically related to almost pure N_2_ fluid inclusions. The presence of the almost pure N_2_ fluid inclusions in these types of localities therefore can be used as a proxy for scheelite precipitation and thus, the presence of W ± Au deposits.

## References

[1] Lehmann MF (2003). Modelling nitrogen and oxygen isotope fractionation during denitrification in a lacustrine redox-transition zone. Geochim. Cosmochim. Acta..

[2] Vigouroux N (2008). Volatiles in high-K magmas from the western trans-Mexican volcanic belt: evidence for fluid fluxing and extreme enrichment of the mantle wedge by subduction processes. J. Petrol..

[3] Van den Kerkhof AM, Kooi ME, Schouten JA, Istrate G, Althaus E (1998). The system CO_2_-N_2_ at high pressure and applications to fluid inclusions. Geochiml. Cosmochim. Acta.

[4] Andersen T, Burke EAJ, Neumann ER (1995). Nitrogen-rich fluid in the uppermantle: fluid inclusions in spinel dunite from Lanzarote, Canary Islands. Contrib. Mineral. Petrol..

[5] Bohlke JK, Ericksen GE, Revesz K (1997). Stable isotope evidence for an atmospheric origin of desert nitrate deposits in northern chile and southern California, USA. Chem. Geol..

[6] Cheilletz A (1988). Stratiform tungsten deposit: a review. Geol Mijinbounw.

[7] Dewaele S (2016). Genesis of the vein-type tungsten mineralization at nyakabingo (rwanda) in the karagwe–ankole belt, central Africa. Mineral. Depos..

[8] Gibert F, Moine B, Schott J, Dandurand JL (1992). Modeling of the transport and deposition of tungsten in the scheelite-bearing calc-silicate gneisses of the montagne noire, France. Contrib. Mineral. Petrol..

[9] Uspensky E, Brugger J, Gräser S (1998). REE geochemistry systematics of scheelite from the Alps using luminescence spectroscopy: from global regularities to facies control. Schweiz. Mineral. Petrogr. Mitt.

[10] Wei P (2008). Single grain zircon shrimp u-pb chronology the geological significance of the wudaogou rock group, yanbian. Geology in China..

[11] Chen C (2014). Permian age of the Wudaogougroup in eastern Yanbian: detrital zircon U–Pb constraints on the closure of the Palaeo-Asian Ocean in Northeast China. Int. Geol. Rev..

[12] Li, D. J. Stratigraphy (Lithostratic) of Jilin Province. China. *Univ. Geosci. Press, Wuhan*. 12–300 (1997).

[13] Chen C (2015). The whole-rock geochemical composition of the Wudaogou group in eastern Yanbian, NE China–new clues to its relationship with the gold and tungsten mineralization and the evolution of the Paleo-Asian ocean. Resour. Geol..

[14] Ren YS, Lei E, Zhao HL, Wang H (2010). Characteristics of fluid inclusions and ore genesis of Yangjingou large scheelite deposit in Yanbian area, NE China. J. Jilin. Univ..

[15] Zhang WB (2007). Geological characteristics of the Yangjingou scheelite deposit of Jilin and its ore genesis. Mineral. Resour. Geol..

[16] Zhao, H. L. Ore Genesis and Geodynamic Settings of Tungsten Deposits in Eastern Jilin and Heilongjiang Province (Eds), **150**, 12–135 (2013).

[17] Hu CT (2010). Geological features and prospecting direction of Yangjingou scheelite deposit, Jilin. Mineral. Explor..

[18] Ren YS (2010). Trace element and rare earth element geochemistry of the scheelite and ore genesis of the Yangjingou large scheelite deposit in Yanbian area, northeastern China. Acta. Petrol. Sin..

[19] Shan CH, Feng LI, Shi JF, Zhao MR (2004). Geological and geochemical characteristics and ore indicator of the Yangjingou scheelite deposit. Mineral. Resour. Geol.

[20] Bakker RJ, Jansen JBH (1993). Calculated fluid evolution path versus fluid inclusion data in the COHN system as exemplified by metamorphic rocks from Rogaland, south‐west Norway. J Metamor Geol.

[21] Burke EAJ (2001). Raman microspectrometry of fluid inclusions. Lithos.

[22] Thiéry R, Van den Kerkhof AM, Dubessy J (1994). vX properties of CH_4_-CO_2_ and CO_2_-N_2_ fluid inclusions: modelling for T < 31 °C and P < 400 bars. Eur. J. Mineral..

[23] Kerkhof Vden, Alfons, Thiery R (2001). Carbonic inclusions. Lithos..

[24] Wopenka B, Pasteris JD, Freeman JJ (1990). Analysis of individual fluid inclusions by fourier transform infrared and Raman microspectroscopy. Geochim. Cosmochim. Acta..

[25] Clayton RN, O’Neil JR, Mayeda TK (1972). Oxygen isotope exchange between quartz and water. J. Geophys. Res. Atmos..

[26] Wen Q (1990). Method of measurement of nitrogen isotope ratio in natural gas. Acta Sedimentologica Sinica..

[27] Bebout GE, Fogel ML (1992). Nitrogen-isotope compositions of metasedimentary rocks in the catalina schist, california: implications for metamorphic devolatilization history. Geochimi. Cosmochim. Acta.

[28] Roedder, E. *Volume 12: Fluid inclusions*. Mineralogical (1984).

[29] Van den Kerkhof AM (1990). Isochoric phase diagrams in the systems CO_2_-CH_4_ and CO_2_-N_2_: Application to fluid inclusions. Geochimi et Cosmochim Acta.

[30] Kerkhof, A. M. V. D. Phase transitions and molar volumes of CO_2_-CH_4_-N_2_ inclusions. *Bulletin de minéralogie* **111**(3–4), 257–266 (1988).

[31] Wood SA, Samson IM (2000). The hydrothermal geochemistry of tungsten in granitoid environments: I. relative solubilities of ferberite and scheelite as a function of T, P, pH, and m Nacl. Econ. Geol..

[32] Dubessy J (1987). Physical and chemical controls (fO2, T, pH) of the opposite behaviour of U and Sn-W as exemplified by hydrothermal deposits in France and Great Britain, and solubility data. Bull. Mineral..

[33] Lin YE, Liu Y, Yang Y, Wei G (2012). The characteristics and significance of pure nitrogen fluid inclusions in Xikuangshan copper deposit, Dongchuan, Yunnan of China. Chin. J. Chem..

[34] Ague JJ (2007). Models of permeability contrasts in subduction zone mélange: implications for gradients in fluid fluxes, Syros and Tinos Islands, Greece. Chem Geol.

[35] Klemd R, Van den Kerkhof AM, Horn EE (1992). High-density CO2-N2 inclusions in eclogite-facies metasediments of the Münchberg gneiss complex, SE Germany. Contri. Mineral. Petrol..

[36] Dong S. M. & Ying, J. L. Geochemistry of tungsten. *Sci. Press*. Beijing (1987).

[37] Bebout GE (1997). Nitrogen isotope tracers of high-temperaturefluid–rock interactions: case study of the Catalina Schist, California. Earth. Planet. Sci. Lett..

[38] Van den Kerkhof AM, Touret JLR, Kreulen R (1994). Juvenile CO_2_ in enderbites of Tromøy near Arendal, southern Norway: a fluid inclusion and stable isotope study. J. metamorphic Geol..

[39] Watenphul A, Wunder B, Wirth R, Heinrich W (2010). Ammonium-bearing clinopyroxene: a potential nitrogen reservoir in the earth’s mantle. Chem. Geol..

[40] Bebout GE, Cooper DC, Bradley AD, Sadofsky SJ (1999). Nitrogen-isotope record of fluid–rock interactions in theSkiddaw Aureole and granite, English Lake District. Am. Mineral..

[41] Zhu XY, Wang JB, Wang YL, Chen XY, Qi B, F. U, Tian Y (2015). The differences of the ore-forming fluid between the vein-type and skarn type tungsten deposits. Acta. Petrol. Sin..

[42] Huang, G. Discussion on genetic relation between the Naozhi gold deposit and the mesozoic volcanic rock series in Naozhi, Jilin province. *Mineral. Resour. Geol*.**1** (1997).

[43] Wang KY, Qing M, Sun FY (2010). Study on the geochemical characteristics of ore-forming fluids and genesis of Xiaoxinancha gold-copper deposit, Jilin Province. Acta. Petrol. Sin..

[44] Duit. W, Jansen JBH, Van, Breemen A, Bos A (1986). Ammonium micas in metamorphic rocks as exemplified by Dome de l’Agout (France). Am. J. Sci..

[45] Jia Y (2006). Nitrogen isotope fractionations during progressive metamorphism: a case study from the Paleozoic coomameta sedimentary complex, southeastern Australia. Geochim. Cosmochim. Acta..

[46] Svensen H (2008). Nitrogen geochemistry as a tracer of fluid flow in a hydrothermal vent complex in the karoo basin, south Africa. Geochim. Cosmochim. Acta..

[47] Peter A, Cawood. C (2007). Buchan. Erratum to “linking accretionary orogenesis with supercontinent assembly”. Earth-Sci. Rev..

[48] Holloway JR (1973). The system pargasite-H2O-CO2: a model for melting of a hydrous mineral with a mixed-volatile fluid—experimental results to 8 kbar. Geochimi. Cosmochim. Acta..

[49] Rafal’skiy RP, Bryzgalin OV, Fedorov PL (1984). Tungsten migration and scheelite deposition under hydrothermal conditions. Geochemistry International.

[50] Busigny V, Bebout GE (2013). Nitrogen in the silicate earth: speciation and isotopic behavior during mineral-fluid interactions. Elements.

[51] Connolly JAD, Cesare B (1993). C‐O‐H‐S fluid composition and oxygen fugacity in graphitic metapelites. Journal of metamorphic geology.

[52] Winter, J. D. An introduction to igneous and metamorphic petrology. Prentice Hall, New Jersey (2010).

[53] Luo ZH, Lu XX, Guo SF (2008). Metallogenic systems on the transmagmatic fluid theory. Acta. Petrol. Sin..

[54] Sun, J. G. *et al*. Ore-forming mechanism for the xiaoxinancha au-rich cu deposit in yanbian, jilin province, china: evidence from noble gas isotope geochemistry of fluid inclusions in minerals. **51**(2), 216–228 (2008).

[55] Wu FY (2011). Geochronology of the phanerozoicgranitoids in northeastern China. J.Asian.Earth.Sci..

[56] Taylor HP (1974). H.P. The application of oxygen and hydrogen isotope studies to problems of hydrothermal alteration and ore deposits. Econ.Geol..

